# The Effect of Opioid Receptor Blockade on the Neural Processing of Thermal Stimuli

**DOI:** 10.1371/journal.pone.0012344

**Published:** 2010-08-27

**Authors:** Eszter D. Schoell, Ulrike Bingel, Falk Eippert, Juliana Yacubian, Kerrin Christiansen, Hilke Andresen, Arne May, Christian Buechel

**Affiliations:** 1 NeuroImage Nord, Department of Systems Neuroscience, University Medical Center Hamburg-Eppendorf, Hamburg, Germany; 2 Department of Neurology, University Medical Center Hamburg-Eppendorf, Hamburg, Germany; 3 Department of Human Biology, University of Hamburg, Hamburg, Germany; 4 Department of Forensic Medicine, University Medical Center Hamburg-Eppendorf, Hamburg, Germany; University of Regensburg, Germany

## Abstract

The endogenous opioid system represents one of the principal systems in the modulation of pain. This has been demonstrated in studies of placebo analgesia and stress-induced analgesia, where anti-nociceptive activity triggered by pain itself or by cognitive states is blocked by opioid antagonists. The aim of this study was to characterize the effect of opioid receptor blockade on the physiological processing of painful thermal stimulation in the absence of cognitive manipulation. We therefore measured BOLD (blood oxygen level dependent) signal responses and intensity ratings to non-painful and painful thermal stimuli in a double-blind, cross-over design using the opioid receptor antagonist naloxone. On the behavioral level, we observed an increase in intensity ratings under naloxone due mainly to a difference in the non-painful stimuli. On the neural level, painful thermal stimulation was associated with a negative BOLD signal within the pregenual anterior cingulate cortex, and this deactivation was abolished by naloxone.

## Introduction

Nociceptive information processing and related pain perception is subject to substantial facilitatory and inhibitory modulation [1]. Inhibitory mechanisms can alleviate pain under certain, often cognitively or emotionally triggered, states [2] such as placebo [3] and stress-induced analgesia [4]. Most importantly, both phenomena point toward the importance of the endogenous opioid system in pain modulation, as indicated by blockade of the effect in the presence of the opioid antagonist naloxone [3], [5], [6]. Although basic pain perception has been a topic of intense interest in functional imaging [7], [8], only more recently have the neuro-anatomical networks underlying pain modulation also been investigated [9]–[16]. The converging evidence from these studies on different cognitive modulations of pain (e.g. attention, hypnosis, anticipation, feeling of control) points to the importance of the anterior medial wall for pain modulation that seems to exert downstream control via subcortical areas such as the amygdala and periaqueductal grey. Interestingly, these activations greatly overlap with brain areas with high opioid receptor density [12], [16].

The aim of this study was to investigate the effect of opioid receptor blockade on pain processing in the absence of any explicit cognitive manipulation. Therefore, we investigated BOLD (blood oxygen level dependent) responses and subjective ratings to painful and non-painful contact heat stimuli with or without the concomitant administration of the opioid receptor antagonist naloxone using a double-blind, cross-over (i.e. within subject) design. Based on previous data [17] we expected higher subjective pain ratings under endogenous opioid receptor blockade (the naloxone session) and a neural effect in areas that are involved in pain processing and have a high density of opioid receptors such as the anterior cingulate cortex (ACC), periaqueductal gray (PAG) and amygdalae (AMG).

## Methods

### Subjects

A total of 20 subjects were recruited from the local community (age: 29.3±4.8 years, right-handed, 10 men). Two subjects did not complete the experiment; one subject had missing data due to a technical failure and one subject had severe movement related artifacts. This left a total of 16 subjects (8 men) for all analyses. All subjects had normal pain thresholds at the site of stimulus application, no history of pain and were not depressed (Beck's Depression Inventory, test scores were 9 or below, mean  = 2.1, standard deviation  = 2.6). All procedures and methods were approved by the local ethics committee, and all subjects gave written informed consent.

### Experimental Protocol

We employed a double-blind, cross-over, counter-balanced design to test for the effect of naloxone on painful and non-painful thermal stimuli. Of the 16 final subjects, 8 received naloxone during the first session. The 2 experimental sessions (identical except for treatment) were one week apart. The subjects were told that on one of the two days they would receive an opioid receptor antagonist named naloxone, which might or might not change their perception of the thermal stimuli. They were not told how their perception might change.

We administered a bolus dose of 0.15 mg/kg naloxone (Naloxon-ratiopharm, Ratiopharm, Ulm, Germany) or saline via an i.v. line inserted into the antecubital vein of the left arm. Because naloxone has a relatively short half-life (∼70 min in blood plasma; Summary of Product Characteristics, Ratiopharm) and its clinically effective duration of action can be even shorter [18], we also administered an intravenous infusion dose of 0.2 mg/kg/h naloxone or saline (diluted in 250 ml of saline), starting shortly after bolus administration. This dosing regime leads to a stable concentration of naloxone in blood plasma over the length of the experiment (see Figure S1) and is sufficient to block central opioid receptors completely [19]. Note that previous studies using either only an equivalent bolus dose [20] or an equivalent bolus dose in combination with a lower infusion dose [10] have observed reliable naloxone effects.

After receiving the bolus, the subject was then led into the scanner. A 30×30 mm thermode (peltier device, TSAII, Medoc, Israel) was placed on the calf of the subject's left leg and an fMRI compatible mouse was placed in the subject's right hand. We did not measure skin temperature; all temperatures reported are those entered into and monitored via the CoVAS program. First, the pain threshold was determined using the method of limits [21]. Thresholds were obtained before and after each session with ramped stimuli (1°C/s starting at a baseline of 32°C and with an upper limit of 52°C to avoid tissue injury). Also before each session, a randomized series of 6 thermal stimuli (43–48°C, plateau duration 6 s) were administered for the subject to practice the rating procedure. All thermal stimuli in the experiment (except when determining threshold) started at a baseline temperature of 32°C and used a ramp rate of 10°C/s. The subjects rated the thermal stimuli using a VAS (visual analogue scale) [22], a bar presented using Presentation (http://www.neurobehavioralsystems.com) and projected onto a mirror atop the head coil. The VAS had an anchor at 0 (“nothing perceived”) and at 100 (“unbearable pain”) with 50 marking the pain threshold. The color of the VAS changed from yellow to red at 50, clearly demarcating the non-painful scale and the pain scale.

**Figure 1:**
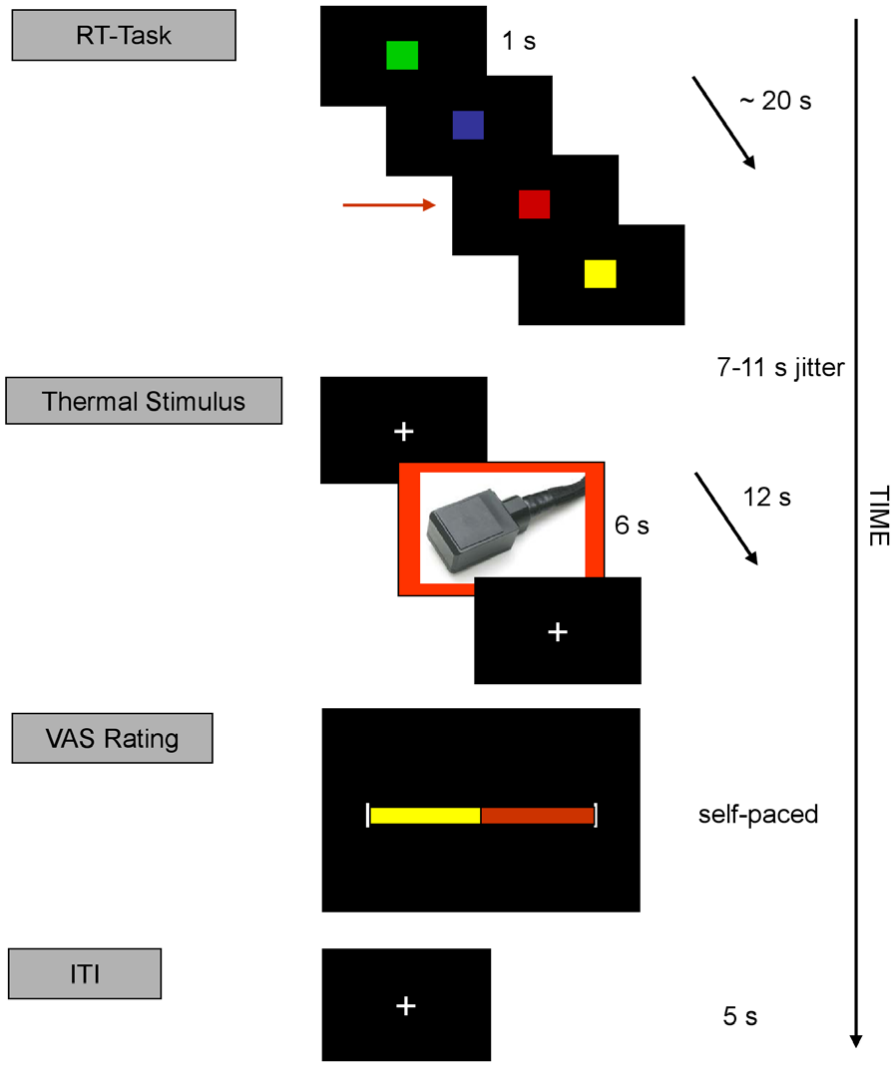
Experimental paradigm. Each trial began with a reaction time (RT) task during which the subject had to press a button whenever a red square appeared (arrow). The squares were presented randomly and appeared for 1 s. Thereafter, cross-hairs appeared and eventually blinked to warn that a thermal stimulus was coming. The blink was sandwiched between a 3–5 s and 4–6 s jitter. Following the second jitter came the thermal stimulus of 6 s (44°C, 45.5°C, 47°C, or 48°C) within a 12 s cross-hair presentation. The visual analogue rating (VAS) scale then appeared, which consisted of two anchors at 0 “nothing” and 100 “intolerable pain” with a third anchor at 50 to mark the pain threshold. The subject could move the edge of the right-hand side of the scale back and forth to the appropriate spot for as long as desired. The starting point of the VAS scale varied randomly for each trial. Once a subject selected a position on the VAS, a cross-hair appeared for 5 s until the start of the next reaction time task.

Each fMRI session consisted of 40 trials and lasted 40–45 minutes. Each trial began with a reaction time task, followed by the thermal stimulus, and ended with the rating procedure, in which the subject rated the perceived intensity of the thermal stimulus just received. The reaction time task, administered to ensure vigilance, required the subject to watch a series of squares in blue, green, yellow and red and press a button whenever the red square appeared. A total of 20 squares for each trial were presented randomly and each square appeared for 1 s. The reaction time task was included to keep the subjects engaged during the long pauses between thermal stimuli, included based on [23]. After this 20 s task, a fixation cross appeared and eventually blinked to indicate the beginning of the thermal stimulus portion of the trial. The blink was embedded between a 3–5 s and 4–6 s jitter. Following the second jitter, a trigger pulse was sent to the thermode to start the thermal stimulus, which had a 6 s plateau. Twelve seconds after the trigger pulse was sent to the thermode, the VAS scale was presented for the subjects to rate. There was no time limit to the rating procedure. This left an average of 62.1 s (standard deviation  = 2.1 s, range 46.5–83.7 s) between consecutive thermal stimuli (Figure 1).

Based on a pilot study, four temperatures were used for the thermal stimuli: 44°C was barely perceptible, 45.5°C was almost at the pain threshold, 47°C was slightly above the pain threshold, and 48°C was definitely above the pain threshold (pre-defined as a VAS score of 50). The pilot study also demonstrated that the perception of pain started 3–4 seconds after the trigger pulse was sent to the thermode. Each of the 4 temperatures was presented 10 times in a pseudo-randomized order that was kept constant within subjects (the subject was presented with the same randomization for both the saline and naloxone sessions). The rating procedure began 12 seconds after the trigger pulse was sent to the thermode. During the thermal stimulation and until the VAS appeared, the subjects saw a fixation cross. After the rating was entered, the fixation cross reappeared for 5 s before the next trial.

### Statistical Analysis of Behavioral Data

All statistical analyses of the behavioral data were done in Matlab. Paired t-tests were used to test for the effect of treatment on the intensity ratings and on general attention (reaction time task). The significance threshold was set to 0.05.

### Image data acquisition and processing

Subjects were scanned with a 3 T Siemens Trio using a T2*-sensitive EPI sequence (TR  = 2.4 s, TE  = 25 ms, flip-angle 80°, FoV 192×192 mm, 3×3×3 mm voxel size) and an 8-channel head-coil. SPM5 (http://www.fil.ion.ucl.ac.uk/spm) was used for all pre-processing and statistical analyses of scans. Pre-processing included slice-time correction to the middle slice, realignment, spatial normalization to a standard EPI template and smoothing with an 8 mm FWHM isotropic Gaussian kernel.

In order to compare the effect of treatment on pain, the thermal stimuli were separated according to VAS rating score. The “non-painful” category contained all stimuli rated 50 and below and the “painful” category contained all stimuli rated above 50, leading to a 2×2 (condition: non-painful and painful by treatment: naloxone and saline sessions) factorial design.

All voxels within the brain were examined with a conventional general linear model-based statistical analysis. For each individual, the design matrix consisted of 5 regressors for each session. The regressors were established by convolving a delta function for the events or a box car for the blocks with the canonical hemodynamic response function as implemented in SPM5. Time and dispersion derivatives were also included for each regressor. The design matrix modeled the following for each session: (1) non-painful stimuli, (2) painful stimuli, (3) anticipation of a thermal stimulus (blinking cross), (4) the 20 s blocks of the reaction time task and (5) the button presses. As mentioned above, the time lapse between trigger pulse and the subjective perception of pain was measured in the pilot study and determined to be about 3 s. The onsets for the non-painful and painful stimuli were therefore calculated as the time of the trigger pulse plus 3 seconds.

Contrasts of interest were set up on the single subject level and entered into a random effects analysis to examine activations across subjects via a one sample t-test. We tested for the main effect of intensity (painful stimuli minus non-painful stimuli), the main effect of treatment (naloxone session minus saline session) and the interaction of treatment and intensity (naloxone(painful – non-painful) - saline(painful – non-painful)).

Correction was based on regions of interest comprising classical pain areas (thalamus, insula, SII, SI and the mid-cingulate region, for a review see [8], as well as areas known to be involved in endogenous anti-nociception (ACC, PAG [12]). Small volume correction was performed with templates constructed from the aal (automated anatomical labeling) toolbox [24], except for the PAG, for which the seed voxel from [9] was taken. All results are reported at p<0.05 corrected for multiple comparisons.

## Results

### Behavioral

The average pain thresholds (± standard deviation) before (pre-session) and after (post-session) each session were 46.7±2.6°C and 49.5±0.8°C for saline and 47.4±2.1°C and 49.7±0.9°C for naloxone (Table 1).

**Table 1:** Summary of behavioral data.

	SALINE		NALOXONE	
	***Pre-session***	***Post-session***	***Pre-session***	***Post-session***
**Thresholds**	46.7±2.6°C	49.5±0.8°C	47.4±2.1°C	49.7±0.9°C
	***Non-painful***	***Painful***	***Non-painful***	***Painful***
**Ratings**	23.3±2.5	73.6±2.4	20.7±2.4	73.6±2.0
**No. of Stimuli**	21±7	19±7	21±6	19±6

Pain thresholds were measured before (pre-session) and after (post-session) the experimental session. The mean and standard deviation are given in degrees Celsius.

The average ratings with standard error are in arbitrary units and categorized as non-painful (VAS < = 50) or painful (VAS >50) for each treatment condition (saline or naloxone). The average total number of stimuli for the particular category with standard deviation for each category is listed under No. of Stimuli.

Under saline, an average (±standard deviation) of 21±7 trials were rated as non-painful (< = 50 on the VAS) and 19±7 trials were rated as painful (>50 on the VAS). Under naloxone, 21±6 trials were rated as non-painful and 19±6 trials were rated as painful (Table 1). The difference between the painful and non-painful average VAS ratings of thermal stimuli was significantly greater in the naloxone session as compared to the saline session (one-tailed, T(15)  = 1.8, p<0.05). It should be noted that this difference is driven by the non-painful stimuli. The average ratings with standard error for each condition are listed in Table 1. Although the intensity ratings of the men tended to be lower, there was no significant effect of gender, neither across nor within treatment sessions.

To check if naloxone had an effect on general attention, we tested performance on the reaction time task (series of colored squares) under naloxone and saline. Neither the reaction times nor the miss rates (percentage of red squares not reacted to) were significantly different across treatment sessions.

**Figure 2:**
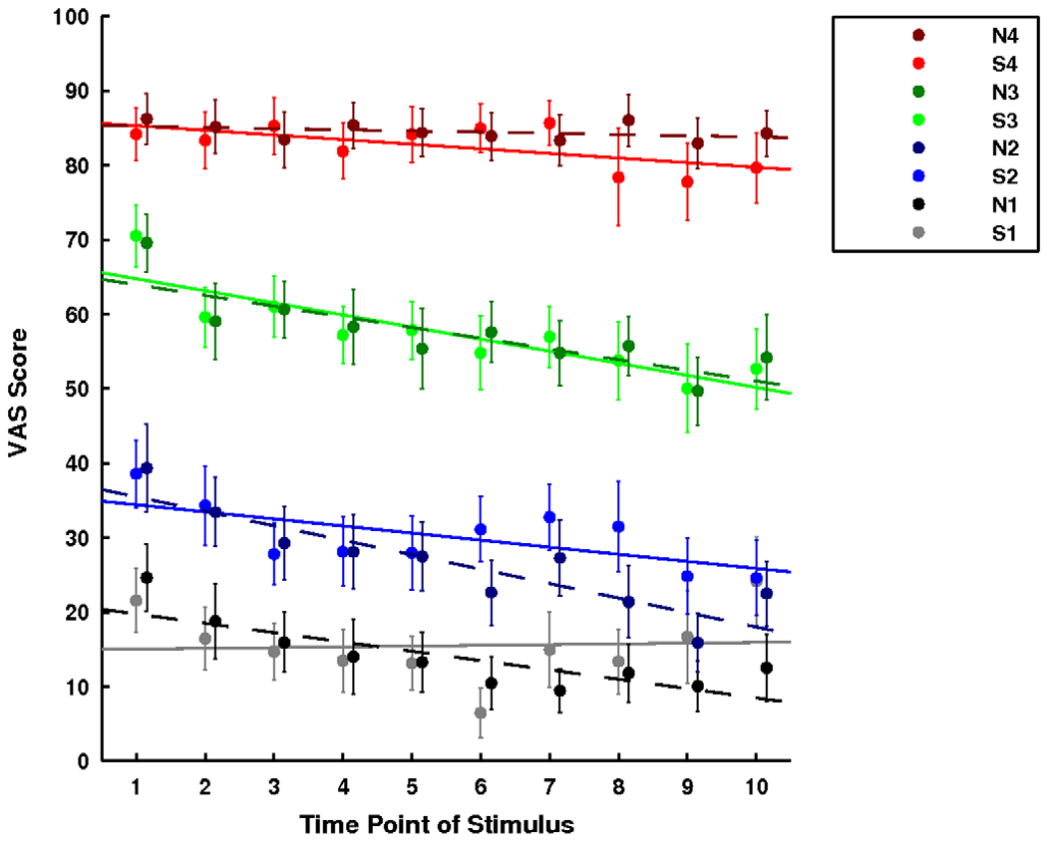
Time course of ratings per temperature. The graph illustrates the average VAS score (± sem) for the ten time points of each temperature. Time point 1 would be the first time that temperature had been presented and time point 10 the last. A capital S indicates the saline session and a capital N the naloxone session. 1 stands for 44°C, 2 for 45.5°C, 3 for 47°C and 4 for 48°C. To test for habituation or sensitization, we compared the average rating over the second half of the session to the average rating over the first half of the session. There was no significant difference for any temperature.

The reaction time task was included in the paradigm to offset habituation and/or sensitization to the stimuli over the course of the experiment [23]. We tested this by comparing the first half of the session to the second half of the session for each temperature within each treatment. There was no indication of habituation or sensitization (Figure 2).

### Imaging

#### Main effect of intensity

**Figure 3:**
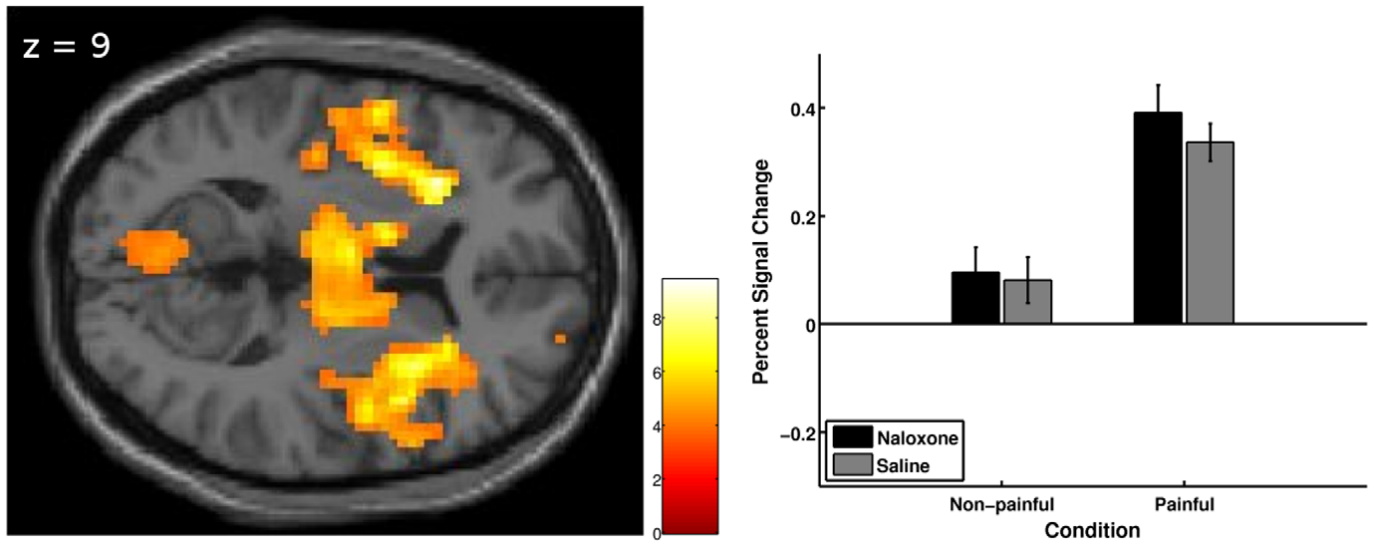
Main effect of intensity on brain activation. Left: Activation (visualization threshold p<0.001 uncorrected) related to painful vs. non-painful stimulus intensities across treatment, overlaid on the axial slice of a T1-weighted template image. The image shows bilateral activation in the insula and thalamus; see Table 1 for a complete listing of results. The color bar represents t-values. Right: Plotted are the percent signal changes (+sem) for the peak voxel in the right insula [33 12 9] for the 2 intensities (non-painful or painful) under each treatment condition (naloxone or saline session). Percent signal change was computed using rfxplot [61].

When comparing painful stimuli to non-painful stimuli across treatment, several areas known to be involved in pain processing showed significant activation. These areas included bilateral insula, bilateral thalamus, bilateral amygdala, bilateral basal ganglia and right (contralateral) periaqueductal gray (Table 2 and Figure 3).

**Table 2:** Imaging results for the main effect of intensity and for the interaction of treatment by intensity.

Region	Coordinates			Z-value voxel level	P-value* SVC corrected
	*X*	*Y*	*Z*		
***Main Effect of Intensity***					
Insula	−30	24	9	4.62	0.001
	33	12	9	4.81	<0.001
	−36	−21	15	4.32	0.004
Thalamus	−6	−9	9	4.52	0.001
	9	−15	0	4.82	<0.001
Amygdala	−18	3	15	5.00	<0.001
	21	−3	−12	4.00	0.002
Caudate Nucleus	−12	6	9	4.27	0.003
	9	3	9	3.56	0.034
Globus Pallidus	−12	3	−3	3.94	0.003
	9	6	−3	3.95	0.003
Putamen	18	12	9	4.20	0.004
PAG	6	−18	−3	4.78	0.001†
***Interaction of Treatment by Intensity***					
Pregenual ACC	−15	42	12	4.14	0.007

Main effect of intensity across treatment thresholded at p<0.001 uncorrected and small volume corrected (at p<0.001) using the AAL-template [24] for the region listed, except for the PAG(†), for which the seed voxel [3 −21 −3] from [9] was used. Treatment (naloxone vs saline session) by intensity (painful vs non-painful rating) interaction. Small volume corrected using the AAL-template of the left anterior cingulate cortex. AAL  =  automated anatomical labeling, SVC  =  small volume corrected, PAG  =  peri-aqueductal gray, ACC  =  anterior cingulate cortex *All p-values listed are family-wise-error corrected.

#### Treatment by intensity interaction

**Figure 4:**
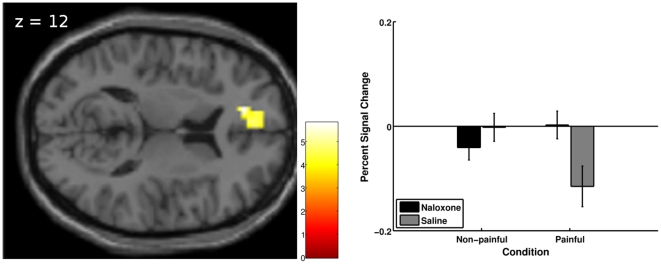
Treatment by intensity interaction. Left: Results (visualization threshold p<0.001 uncorrected) related to the treatment (naloxone vs. saline session) by intensity (painful vs. non-painful) interaction, overlaid on the axial slice of a T1-weighted template image. The image shows the cluster around the peak voxel in the pregenual ACC. The color bar represents t-values. Right: Plotted are the percent signal changes (+sem) for the peak voxel [−15 42 12] for the intensities under each condition. Percent signal change was computed using rfxplot [61].

To test for a differential response to thermal intensity dependent on treatment, we set up the contrast (naloxone(painful – non-painful) - saline(painful – non-painful)). The interaction analysis revealed a significant effect in the anterior cingulate cortex (pregenual ACC: peak voxel in cluster [−15, 42, 12], Z  = 4.14, cluster size  = 61, p = 0.007 small volume FWE corrected, Table 2 and Figure 4 left). Specifically, under physiological conditions (saline), a negative BOLD response to painful stimulus intensity was observed, which was significantly reduced by the administration of naloxone (Figure 4 right).

**Figure 5:**
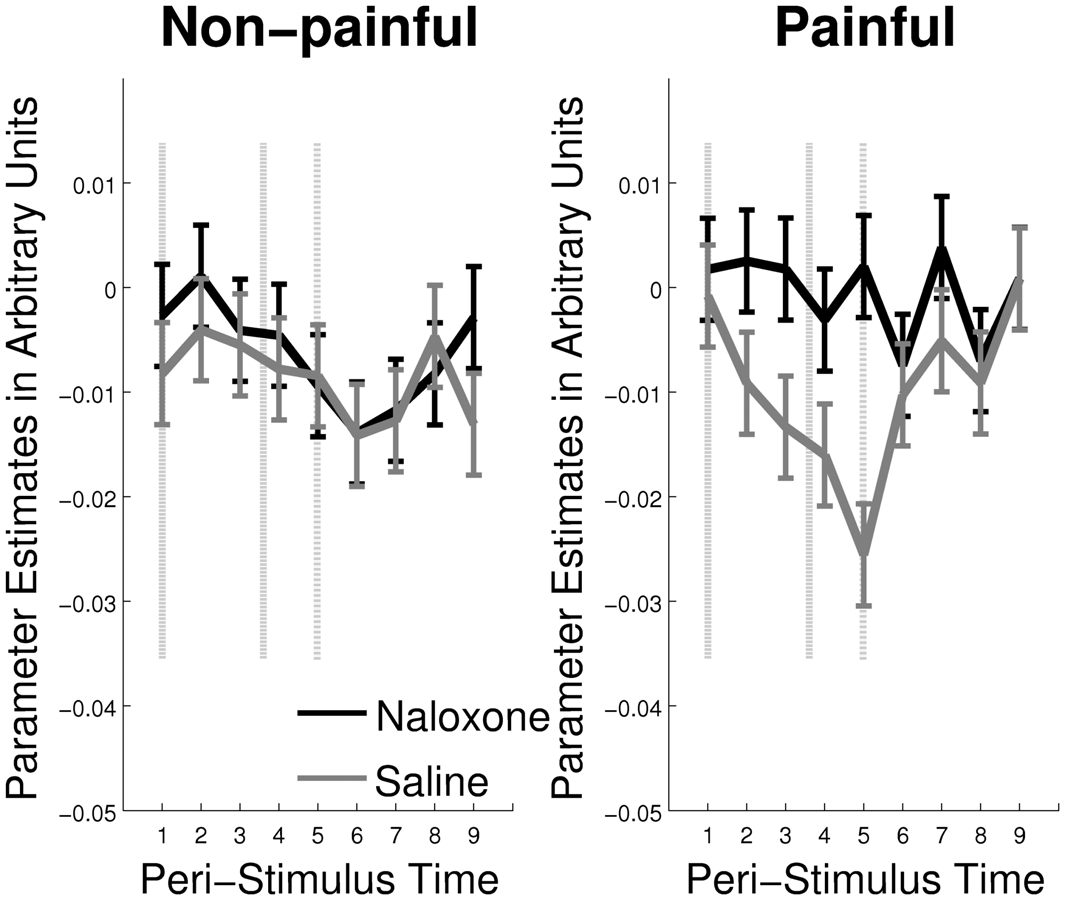
FIR Analysis of the BOLD response in the pgACC. Finite Impulse Response (FIR) analysis of the BOLD response to non-painful and painful thermal stimulation in the pregenual ACC for the peak voxel from the interaction analysis [−15 42 12]. Peri-stimulus time is in scans. The dashed lines demarcate the beginning and end of the thermal stimuli, including ramp-time (mean length ± std, 3.66±0.14 scans). The dash-dot line demarcates the beginning of the VAS rating procedure.

To further characterize the BOLD signal, we ran a finite impulse response (FIR) analysis on the 12 second period between thermal stimulus onset and rating procedure onset. Figure 5 shows the negative BOLD signal under saline for the painful stimuli that is blocked by naloxone for the peak voxel from the interaction of treatment and intensity analysis [−15, 42, 12].

## Discussion

To investigate the role of the endogenous opioid system in physiological pain processing, we combined painful and non-painful thermal stimuli with a pharmacological intervention using the opioid antagonist naloxone in a double-blind, cross-over design. On the behavioral level, this led to an increased difference between painful and non-painful ratings under naloxone; this difference was driven by the difference in the non-painful ratings. Functional neuroimaging revealed that painful thermal stimulation leads to a negative BOLD signal within the pregenual ACC, which is blocked by the administration of naloxone, suggesting an inhibitory influence by endogenous opioids in this region.

Studies on opioid blockade in healthy human subjects have yielded mixed results. Several studies do not find an effect of naloxone on pain using either painful shocks [25], limb ischemia [26], [27] or cold-water immersion [26]. Most recently, Kern et al. [28] studied the paradoxical thermal-grill, as well as heat- and cold-induced pain. They found no effect of naloxone on any (heat, cold, paradoxical) pain ratings.

However, other studies have successfully induced hyperalgesia with naloxone. Earlier studies used dental post-operative pain [6], [29], [30] or electric shocks [31], whereas a more recent study used a combination of capsaicin and naloxone [32]. Yet another study specifically targeted the mechano-insensitive nociceptors via transdermal electrical stimulation and concluded that it is not necessarily the magnitude of the perceived pain that is needed for endogenous opioid release, but rather the activation of the mechano-insensitive nociceptors [33]. This suggests that longer and more intense pain stimuli more easily activate the opioid system, as these conditions are also more likely to activate the mechano-insensitive nociceptors. However, aside from the elegant studies by Koppert [33], [34], no studies systematically examine naloxone sensitive and naloxone insensitive pain using one type of stimulus and dosing regimen.

In this study, the difference in intensity ratings between saline and naloxone is driven by lower ratings for the non-painful stimuli under naloxone. This is contrary to our hypothesis as we expected higher intensities for the painful stimuli under naloxone to drive the difference. To our knowledge, none of the studies that have looked at non-painful thermal stimuli under naloxone and saline have found a significant difference [28]. Interestingly, a study looking at the effects of epidural morphine on somatosensory functions found that the warm detection threshold was increased by morphine; this effect was naloxone reversible [35].

We were interested in testing whether naloxone had an effect on intensity ratings in general and on pain intensity ratings in particular, in the absence of any cognitive or affective modulation. The lack of a significant difference in pain intensity ratings in both this within-subject design as well as in the control condition of a between-subject placebo analgesia study by Eippert et al. [36] might indicate that a cognitive/affective modulation is necessary for naloxone to have a behavioral effect. However, there are several studies showing an effect without such modulation. Borras et al. [37] found an effect of naloxone on both pain and intensity ratings during the latter half of a 24 s mild thermal stimulus, Anderson et al. [32] found an effect using capsaicin coupled with thermal stimuli and Koppert et al. [34] found an effect using electrically induced pain. This indicates that there are several factors which may lead to an effect of naloxone on subjective ratings, including stimulus length, stimulus type as well as cognitive/affective modulation.

Only one other study has looked at the effect of endogenous opioid activity and pain on CNS activity through the use of naloxone with fMRI [37]. By using long lasting thermal stimuli (25 seconds at 46°C), this study analyzed both behavioral and imaging data as part of either an “early phase” (first 12 s) or a “late phase” (last 12 s). Across the total of four 46°C thermal stimuli, they report a significant increase in the pain (intensity and unpleasantness) ratings under naloxone only for the late phase. It is also in the late phase that they find a difference between naloxone and saline in the pregenual ACC and insula for mild thermal pain.

Borras et al. suggest that the second of the two-peaked BOLD response (the late phase) represents regions involved in emotion and that these are the regions affected by endogenous opioids. Our design (phasic stimuli including both painful and non-painful intensities) allows us to more cleanly delineate the neural response. We are thereby able to show the influence of the endogenous opioid system and the direction of activation in the pregenual ACC due to increasing thermal intensity.

Our data showed a pain related deactivation of the rACC that was blocked by the administration of naloxone, strongly suggesting an activation of the endogenous opioid system. Consistent with our finding, using opioid ligand PET, Sprenger et al. [38] were able to show a decrease in opioid receptor binding after thermal pain stimuli in the rACC, providing direct evidence for the involvement of this region in the endogenous opioid inhibition of pain.

The rACC is strongly involved in the modulation of pain under the control of cognitive strategies such as attention and placebo analgesia. This region has also been characterized as showing a high concentration of opioid receptors [39] and having a major impact on opioidergic pain modulation [40]. Our data, implying opioid release in the pregenual ACC coincident with painful thermal stimulation, is therefore in line with these reports. The rACC, however, is also closely linked to anxiety states [41] and naloxone may be associated with an increase in anxiety and stress levels [42], [43]. Future studies with naloxone should consider including measures of anxiety and stress.

We observed a distinct negative rather than positive BOLD signal. This observation is in line with a recent fMRI study on placebo analgesia that was able to dissociate areas that were either activated or deactivated under the placebo as compared to the control condition [36]. In agreement with our data, the neural response to placebo in the pregenual ACC, and not the activation in the subgenual ACC was most strongly modulated by naloxone. In addition, this placebo analgesia-induced deactivation was observed during the early and not the late phase of the 20 s painful thermal stimulation, which is in agreement with the stimulus duration of the thermal stimulus used in this study (6 s). In line with these findings, a similar opiate dependent deactivation of the ACC was observed in a study looking at exogenous opiate administration without concomitant pain [44].

Opioid receptors are generally considered inhibitory receptors and one could assume that binding of inhibitory receptors leads to deactivation; however, molecular studies in rats show that opioid receptor binding can lead to both inhibition and excitation. For example, tonic inhibition courtesy of GABAergic-neurons can in turn be inhibited by enkephalinergic neurons, leading to post-synaptic excitation in the periaqueductal gray [45]. Concerning direct inhibition, opiate administration leads to a decrease in extracellular glutamate in the ACC [46] as well as in the PFC [47]. The decrease in glutamate, an excitatory inhibitor, was in turn related to a decrease in neuronal firing in these studies. Constellations of receptors and neurotransmitters are highly heterogeneous between different anatomical locations [45] and the binding of various agonists do not parallel each other [48]. In addition, exogenous opiates and endogenous opioid peptides differ at the molecular level leading to variant cellular processes and finally systems level effects [49].

Recent investigations using fMRI have been able to show that BOLD deactivations are tightly coupled to neuronal activity [50], [51]. Given that opioid receptors function via inhibitory mechanisms [52], it is interesting to compare this system with the effect of other inhibitory neurotransmitters such as GABA. A recent functional neuroimaging study of the GABAergic system has revealed negative BOLD signal changes in the pregenual ACC in humans [53]. In light of these investigations, our finding of a decrease in signal in conjunction with painful stimulus intensities is likely due to opioidergic activity and fits in well with the identification of opioid receptors as inhibitory receptors. Considering the close interaction between the GABAergic and opioidergic systems [54], it is also possible that the deactivation may be directly mediated by GABA.

However, it should also be noted that some, mostly PET studies have also reported opiate dependent activations in the ACC [9], [12], [55]–[60]. Apart from the differences in stimulation and imaging technique, one reason for this discrepancy might be related to low spatial resolution in some studies, which would collapse signals from functionally distinct ACC subareas. This notion is supported by a recent study using considerably higher spatial resolution fMRI and showing opiate dependent activation and deactivation in neighboring ACC subregions during the same condition [36].

In conclusion, our data reveals that the endogenous opioid system is affected by thermal stimuli in the absence of any specific cognitive manipulation. The hypothesis that endogenous opioids lead to a deactivation of the pregenual ACC is supported by our data showing that this effect can be blocked by the opioid receptor antagonist naloxone.

## Supporting Information

Figure S1 Naloxone plasma concentrations: mean (+SEM) over the 4 pilot subjects. Naloxone has a half-life of about 1 hour in man [1]. Since the experiment lasted 1 hour, the concentration at the end of the experiment would have substantially deviated from the concentration at the beginning of the experiment had only a bolus dose been given. Based on [1] and [2], the following parameters were entered into AutoKinetic v3.4b, an MS-Excel-based software for determining dosing strategies: one-compartment model, the individual weight, a half-time of 1.1 h, distribution volume of 2 L/kg. To keep the plasma concentration of naloxone at 50 ng/ml, a dosing strategy of a bolus of 0.15 mg/kg followed by 0.00347 mg/kg/min infusion was suggested. We ran a pilot study with 4 men to test the strategy. References 1. Goldfrank L, Weisman RS, Errick JK, Lo MW (1986) A dosing nomogram for continuous infusion intravenous naloxone. Ann Emerg Med 15: 566–570. 2. Baselt RC (2004) Disposition of Toxic Drugs and Chemicals in Man, 7th Edition. Foster City: Biomedical Publications. 802 p.
(1.07 MB TIF)
