# Supplementary figures and images for: The Effect of Opioid Receptor Blockade on the Neural Processing of Thermal Stimuli
Source: PLoS One. 2010 Aug 27;5(8):e12344. doi: 10.1371/journal.pone.0012344 (PMC2930255; doi:10.1371/journal.pone.0012344)

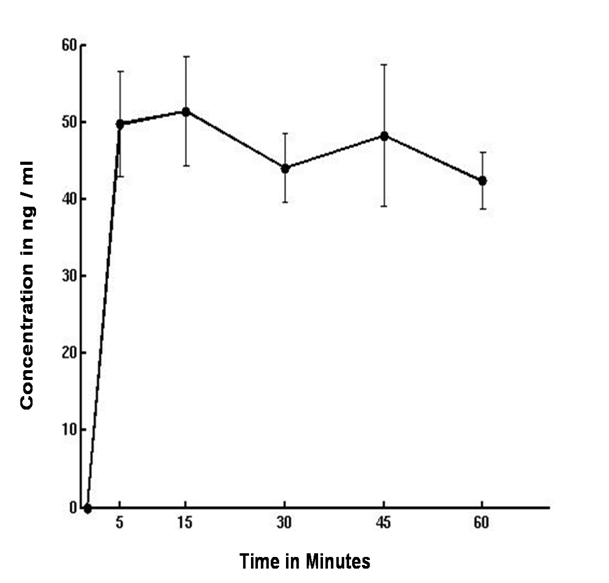

Supplement: Figure S1 — Naloxone plasma concentrations: mean (+SEM) over the 4 pilot subjects. Naloxone has a half-life of about 1 hour in man [1]. Since the experiment lasted 1 hour, the concentration at the end of the experiment would have substantially deviated from the concentration at the beginning of the experiment had only a bolus dose been given. Based on [1] and [2], the following parameters were entered into AutoKinetic v3.4b, an MS-Excel-based software for determining dosing strategies: one-compartment model, the individual weight, a half-time of 1.1 h, distribution volume of 2 L/kg. To keep the plasma concentration of naloxone at 50 ng/ml, a dosing strategy of a bolus of 0.15 mg/kg followed by 0.00347 mg/kg/min infusion was suggested. We ran a pilot study with 4 men to test the strategy. References 1. Goldfrank L, Weisman RS, Errick JK, Lo MW (1986) A dosing nomogram for continuous infusion intravenous naloxone. Ann Emerg Med 15: 566–570. 2. Baselt RC (2004) Disposition of Toxic Drugs and Chemicals in Man, 7th Edition. Foster City: Biomedical Publications. 802 p. (1.07 MB TIF) [file pone.0012344.s001.tif]
